# Adolescent girls’ migration and its impact on early marriage: Qualitative findings in Mali

**DOI:** 10.1371/journal.pone.0230370

**Published:** 2020-03-20

**Authors:** Sarah Engebretsen, Mouhamadou Gueye, Andrea J. Melnikas, Sékou Fofana, Bourama Fané, Sajeda Amin

**Affiliations:** 1 Sarah Engebretsen LLC, Cos Cob, Connecticut, United States of America; 2 Centre d’Etude et de Recherche sur l’Information en Population et en Santé (CERIPS), Bamako, Mali; 3 Population Council, New York, New York, United States of America; Middlesex University, UNITED KINGDOM

## Abstract

Adolescent girls in West Africa are migrating in search of educational and livelihood opportunities. In Mali, early marriage (before the legal age of 16) is a common practice. This paper builds on prior research on female migration that focused on the direct influences of migration on marriage and explores the wider social impact of rising female migration in sending communities by examining direct and indirect effects and intended and unintended consequences. This study examines perceptions about migration among girls and their parents including how it influences marital timing, marriage preparations, marriage practices, and marital relations. Qualitative data were collected from 140 adolescent girls and 115 parents of adolescent girls in rural areas in focus group discussions (FGDs) (n = 31) and in-depth interviews (IDIs) (n = 41) to inform how girls’ migration patterns might influence program recruitment strategies and content for an intervention aimed at addressing early marriage in Mali. Our findings concur with earlier studies that migration has direct effects on marriage because it allows girls to both avoid early marriage and prepare for marriage through the assembly of goods and wares to bring to their conjugal homes. Despite some of the perceived risks of migration on marriage, the indirect effects of migration include allowing girls to see different types of marriage practices and marital relationships between husbands and wives and potentially allowing migrant girls to exert more influence over the marital process compared to non-migrants. However, migration can expose girls to new ideas and alternatives that may be incongruent with cultural expectations for them once they return to their communities. This study suggests that migration is seen as an inevitable part of life for many adolescent girls in Mali. Girls who migrate may return to their villages with not only items or income that provide direct benefits to a marriage, but also viewpoints on the expectations for women and girls in their communities that indirectly influence marital relationships. Although this can be challenging for individual returned girls in terms of reintegration, these new expectations may, over time, lead to social changes that influence migrants and non-migrants. Program strategies and approaches must consider the possibility of migration as an important aspect of every adolescent girl’s opportunity structure. The qualitative data suggests that certain skills are critical for adolescent girls. Programs should emphasize the acquisition of relevant skills such as communication, risk assessment, negotiation and money management in ways that are relevant for migrants and non-migrants.

## Introduction

*Girls who leave in mass [*exode*] bring back things we do not find here*

Focus group, fathers, Sikasso

Migration is transforming the world and driving socioeconomic change in rural communities [1]. Demographers estimated that by the year 2020, most developing countries would have more people living in cities than in rural areas [2]. West Africa has seen an increase in rural to urban migration among women including adolescents [3]. Migrant girls are moving in ever-greater numbers in search of education and livelihood opportunities or to avoid hardship at home [1, 2]. Despite migration becoming a part of life for rural adolescent girls in Mali [1], there is a dearth of evidence on the practice and its influences on the timing of marriage.

In Mali, early marriage, or marriage before the legal age of 16 for girls, is a common practice and child marriage prevention has recently become an important part of the development agenda. Although the Demographic and Health Surveys (DHS) conducted between 1987 and 2012 suggest that the proportion of women who were married before the age of 18 has declined from 80.9% to 50.1% in Mali [4], Mali ranks among the top 5 countries in terms of high rates of child marriage [5], or marriage before age 18. Important policy documents have noted that child marriage is more common in rural areas and government and implementing organizations have located their programmatic efforts accordingly [6]. However, for programmatic purposes, given the salience of migration it is important to understand the extent to which adolescents and parents in rural areas perceive adolescent migration to impact marital timing, marriage preparations, marriage practices, and marital relations.

Adolescent migration in Mali is driven in part by larger trends in GDP growth [7], increased rural-urban migration amidst climate change and environmental degradation [8] and increased international out-migration and remittances. Despite these trends, Mali remains predominantly rural with about 70% of the population living in rural areas. Although agriculture accounts for just over one-third of Mali’s total GDP, close to 80% of the country’s rural population is employed in this sector. Thus limited economic opportunities in rural areas and expansion of opportunities in urban areas through rapid growth of cities have fueled rural to urban labor migration.

As in many parts of the world, there is convincing evidence that independent labor migration by women, motivated primarily by opportunities for work rather than for marriage or other reasons, is common in West Africa [1]. Female migration has been shown to be associated with increases in women’s financial and social empowerment and reductions in familial poverty [3]. Previous research suggests that migration is an important experience for adolescent girls in parts of Mali [1]; however, that research did not focus on the relationship between migration and marriage in particular.

Other studies have explicitly examined migration and some aspects of marriage in Mali. Hertrich and Lesclingand [9] examined the role of migration in Mali’s nuptiality transition and found that migration not only postponed marriage, but also influenced the marriage formalization process by shifting preferences away from traditional and religious marriage arrangements in favor of civil ceremonies. Hertrich and Lesclingand [10] also suggest that skills acquired during migration may color previous impressions of acceptable futures and may be useful for girls in managing their conjugal lives. They find that migration for female adolescents is often viewed as a personal experience to better oneself and often viewed as risky by elder generations [10].

Female migration in Mali is also explicitly tied to preparation of the *trousseau*, or the collection of a bride’s personal possessions usually including clothes, accessories, and household linens and wares that she needs to have assembled before marriage can take place [9, 11]; and the composition of the *trousseau* has important implications for signaling one’s status [12]. Others have examined marriage and migration with regards to marital practices. Marcoux et. al. found that practices around marriage ceremonies differ in urban areas in Mali compared to rural areas [13]. Lardoux reported similar findings, noting that urban areas in Mali have more diversity in type of marital ceremonies than rural areas [14]. Most of these studies explore the connection between migration and marriage by either examining how migrants differ in marriage behavior from non-migrants or how marriage and migration decisions are directly linked.

Female migration in Mali is also an important part of kin relations, as female migrants make connections and support one another in ways that are different from men and have lasting effects on social networks and connectivity [11]. Previous research notes the importance of familial and social relationships in helping women and girls navigate migration to urban areas and in fostering subsequent migration among other kin [15]. Among the limited research on adolescent migration’s impact on timing of early marriage more broadly, Temin et. al. [2]’s secondary analysis of DHS data found that the majority of urban in-migrant girls move before marriage, and Grosz-Ngate [11] found that rural-to-urban migration may allow girls in rural Mali to avoid an undesirable marriage. Grosz-Ngate also noted the shame a girl may bring to her family if she becomes pregnant while working in the city [11].

Building on research on female migration [1,9, 10], migration and marriage timing [10, 11], and marriage practices [14], this paper focuses on migration sending areas to examine indirect effects and unintended consequences of migration on marriage. The paper provides recent evidence of girls’ experiences with migration and community (parents and girls) perceptions of migration including how migration can influence four aspects of marriage: marital timing, marriage preparations, marriage practices, and marital relations. To the best of our knowledge, this paper is the first to document migration’s wider impact on all four related aspects of early marriage in order to inform interventions to delay marriage. Moreover, some of the cited studies were collected from villages when girls first started migrating, whereas this study was conducted at a time when migration had become the norm. Mali experienced rapid urban growth from 2005 to 2015 and is estimated to welcome 400,000 new urban residents per year until 2030 [16]. Given how age at marriage has declined and urban growth and migration have evolved over this time period, we felt it necessary to generate new data on the topic. Although there are numerous migration trajectories, this paper focuses on migration from rural areas in Segou and Sikasso to larger metropolitan areas, frequently Bamako.

### The study area

The study villages are located in Mali’s southeastern region of Segou and southern region of Sikasso. The regional town centers of Segou and Sikasso are 235 km and 370 km away from the capital Bamako, respectively. The Bambara constitute the dominant ethnic group in the Segou study area and the Senoufo/Minianka are the dominant ethnic group in the Sikasso study area [17]. Both regions are in semi-arid areas and rely on rain-fed agriculture with some irrigation from the Niger and Bani Rivers [18]. Education for women in these areas is low: 72.3% of women in Segou and 68.8% in Sikasso reported no formal schooling according to DHS data. Marriage typically occurs before or around age 18: the median age at first union is 18.1 in Segou and 17.2 in Sikasso.

In baseline research (2016) associated with this project [17], we found that in Sikasso 15.4% of girls 12–19 were ever married compared to 14.2% in Segou. Based on midline data for this project [19], we found that movement is common and begins early: 43.0% of girls 12–19 had reported moving outside of their village for three months or more with a mean age at first move of 12.1 years. Socio-demographic characteristics of girls in the baseline sample are presented in Table 1.

**Table 1:** Select Socio-demographic characteristics of girls from baseline survey, 2016 (n = 855).

	Sikasso	Segou
	N = 300	N = 555
**Age (mean)**	15.1	15.3
**Respondents who were currently married (%)**	15.4	13.9
**Respondents who were ever married (%)**	15.4	14.2
**Age at marriage (mean)**	16.1	16.1
**Parental Education**		
**Mother has no formal schooling (%)**	91.1	85.3
**Father has no formal schooling (%)**	79.2	75.6
**Has a national ID card**	63.0	60.3
**Is non-Muslim (%)**	23.5	4.3
**Number of siblings (mean)**	5.3	5.0

## Methods

### More than Brides Alliance (MTBA) intervention and quantitative study

In March 2017, qualitative data were collected as part of a research study examining the More than Brides Alliance (MTBA) intervention. This holistic intervention, led by Save the Children Netherlands, aims to address early marriage through multiple strategies including: empowering at-risk and already married adolescents with life-skills education, comprehensive sexuality education, and sexual and reproductive health and rights information; providing alternatives to early marriage through enhancing access to education, economic opportunities, and child protection systems; increasing sexual and reproductive health services; changing social norms; and influencing legal and policy frameworks. In Mali, the MTBA intervention is implemented by Oxfam Novib and Save the Children. The MTBA intervention began implementing in communities in early 2017 and initial activities included meeting with community leaders and conducting community meetings to explain the program. Activities with adolescent girls directly, such as girl groups or clubs, did not start until late 2017/early 2018, though we acknowledge girls may have been part of larger community activities focused on raising awareness about early marriage.

As the research and evaluation partner, the Population Council is collecting quantitative data [17, 19, 20] to assess program impact on age at marriage as well as qualitative data to explore specific contextual factors influencing early marriage in these communities. While the intervention is not explicitly designed for migrants, the baseline survey (July-August 2016) shed light on experience of migration among adolescent girls and raised questions about the influence of adolescent migration on early marriage. Moreover, with survey design and data collection timelines cognizant of migration patterns in the region, qualitative inquiry was a logical next step to understand how migration patterns might influence program recruitment strategies and intervention content. Accordingly, qualitative data were collected in March 2017; baseline respondents were eligible to participate in girl-focused IDIs and FGDs in addition to focus groups designed specifically for parents of adolescent girls. Qualitative data explored the relationship between perceptions and experiences of adolescent migration and early marriage timing, preparations, practices, and marital relations. This paper presents complementary findings on individual perspectives derived from the IDIs alongside norms derived from the FGDs.

### Recruitment and data collection

Qualitative data collection was carried out by *the Centre d’Etude et de Recherche sur l’Information en Population et en Santé* (CERIPS), an experienced social science research group, based in Bamako. Four female research assistants with experience in qualitative data collection were trained over a period of six days in March 2017. Interviewer training covered qualitative methods and limitations, a review of the study instruments, methods for accurate translation and transcription of data, and processes for obtaining informed consent/assent from study participants.

Study sites were selected using baseline data to identify communities with the highest proportion of ever married 12–19 year old girls. One village was selected in each of the areas covered by the MTBA project including three in Segou (Segou/Sekoro, Bla/Soké, and San/Dougouolo) and two in Sikasso (Yorosso/Boura and Sikasso/Tapérela) so that findings from this research could inform program implementation. While the MTBA intervention is implemented in select communities, we did not select girls to participate in the qualitative data collection based on their exposure to the intervention. We recruited individuals who met selection criteria for participation in one of four data collection activities: FGDs with parents of adolescent girls ages 12–19 with mother and father groups conducted separately; FGDs with adolescent girls ages 12–19, conducted separately for unmarried, engaged, and married girls; IDIs with married girls ages 16–19; and IDIs with unmarried girls ages 12–19 (including girls who were engaged to be married). This study sought to gather perspectives from those at home in rural areas rather than focusing on those in receiving communities. Thus our sample includes adolescent girls who did not move, those who had moved and returned to their village, and the parents of girls who were currently away or had previously migrated, though we did not explicitly sample based on these criteria. More detail on the number of data collection activities and participants is provided in Table 2. We identified respondents eligible to participate with the assistance of community leaders and program staff affiliated with a local NGO. We used criterion sampling to create homogenous samples [21] of individuals according to age, marital status, and residence within the community. Parental permission and informed consent procedures were followed prior to including individuals in the research.

**Table 2:** Overview of qualitative data collection.

**In-depth interviews**	Adolescents ages 12–19	24	
	Married adolescents ages 16–19	17	
	**Total number of in-depth interviews**	**41**	
**Focus group discussions**		Number of FGDs	Number of individuals
	Unmarried adolescent girls	5	30
	Married adolescent girls	5	35
	Engaged adolescent girls	5	34
	Fathers of adolescent girls	8	52
	Mothers of adolescent girls	8	63
	**Total number of focus group discussions**	**31**	
	**Total transcripts**	**72**	

### Qualitative instruments, transcription, and translation

Semi-structured interview guides and focus group discussion guides were written in French but conducted with each respondent group in Bambara. In the few cases where respondents did not speak Bambara, a translator was used. The format of the focus group discussions and in-depth interviews was open-ended, facilitating a free flow of ideas from the respondents. Separate guides were designed for each respondent category with related though distinct questions on topics such as the influence of migration on marital timing and experience as well as community perceptions of migrant girls.

The four research assistants who led the qualitative data collection and three additional research assistants transcribed the in-depth interviews (n = 41) and focus group discussions (n = 31) and translated them from Bambara to French.

The study protocol was reviewed and approved by the Population Council’s Institutional Review Board and the Mali-based *Institut National de Recherche en Santé Publique* (INRSP)’s Ethics Committee in 2016.

### Qualitative data analysis

The research team used an iterative approach, identifying relevant themes, developing an initial codebook, and refining themes as analysis progressed. First, the primary coder annotated the text by writing notes in the margins of all 72 transcripts. Then, the primary coder sorted the annotation list into smaller categories and subcategories, eliminated redundancies, and created a preliminary hierarchy [22]. The coder generated a list of code descriptions when thematic categories were not self-explanatory. A team of researchers in Mali and the United States, along with program implementers based in Mali and the Netherlands, refined the list of themes. Final codebook refinement consisted of adding sub-codes, with a total of 21 parent codes. The main themes that emerged from the data related to this analysis include: reasons for migration decisions, work and living arrangements in the city and influence of the city on the girl, the influence of migration on marital timing and experience, and perceptions of migrant girls in their home communities.

The second step in the data analysis process was establishing intercoder reliability, which can be used as a proxy for the validity of constructs emerging from qualitative data [22]. To assess intercoder reliability, two coders were independently given 14 random excerpts of text from a subset of transcripts and the same set of codes (n = 6). Coders were instructed to independently delineate text units and assign codes. Using Dedoose software (version 8), coders achieved a Cohen’s Kappa coefficient of 0.873 for code application, indicating strong agreement among coders. Having obtained an acceptable level of intercoder agreement, the team indexed all 72 transcripts in Dedoose using the agreed-upon themes. The team used memos to record ongoing observations and code reports were run on each code pertinent to the current analysis.

## Results

A total of 255 people participated in qualitative data collection including adolescent girls and parents of adolescent girls. This included 99 girls (ages 12–19) in the FGDs, 17 married girls (ages 16–19) in the IDIs, 24 unmarried girls (ages 12–19) in the IDIs, 52 fathers in FGDs, and 63 mothers in FGDs. We conducted separate focus groups for mothers and fathers that ranged in size from six to ten participants. Adolescent girl focus groups ranged in size from six to nine participants. Tables 3 and 4 provide additional information about the sample included in the research. While migrant status came out in interviews and FGDs, we did not collect information on migrant status at the time of data collection and collected more general demographic information. Where available, we report migration status for select quotations. Compared to our baseline data, the qualitative sample of adolescent girls was a bit older on average (mean of 16.8 in Sikasso and 16.7 years in Segou, compared with mean ages of 15.1 years in Sikasso and 15.3 years in Segou at baseline). These differences are due, in part, to our selection of girls based on marital status, with more (slightly older) married girls included in the qualitative research compared with the baseline study.

**Table 3:** Select Socio-demographic characteristics of adolescent girls in FGDs and IDIs (N = 140).

		FGDs	IDIs
		*N = 99*	*N = 41*
**Age (mean)**		16.8	16.7
**Married: N (%)**		35 (35.3)	18 (43.9)
*Among married*, *type of union*	Monogamous	*Data not available*	11 (61.1)
	Polygamous	*Data not available*	6 (33.3)
	Not specified	35 (100.0)	1 (5.6)
**Engaged**		31 (31.3)	7 (17.1)
*Among engaged*, *who chose*	Own choice	30 (96.8)	4 (57.1)
	Promised by family	1 (3.2)	3 (42.9)
**Never married**		33 (33.3)	16 (39.0)

**Table 4:** Characteristics of parents in FGDs (N = 115).

**Female** (%)		54.8
**Age** (mean)		46.6
**Number of children** (mean)		6.9
**Number of daughters 10–19** (mean)		1.8
**Has a daughter 10–19 ever married or engaged** (%)		48.7

### Migration allows girls to both avoid early marriage and prepare for marriage

Building on earlier research showing that adolescent girls tend to migrate before marrying, this study focused on the influence of migration as an important element of the social context on marriage timing and practices. Some responses, like the one below from an engaged girl in response to a question about migration’s possible impact on marriage, suggested that migration away from the village is directly related to marriage and viewed as an alternative to early marriage:

*For those girls who do not like early marriage*, *they leave and hide out in Bamako for a period of time*.

Focus group, engaged girls ages 12–19, Segou

By offering the opportunity for girls to “hide out”, migration was perceived by adolescent girls to be influential on delaying marriage. This perception was echoed by parents, through illustrative phrases such as “girls escape to go the city frequently to avoid marriage” (Focus group, mothers, Sikasso), though with less frequency than among adolescent girls themselves. However, as shown in the following quotation from a focus group with fathers, marriage delays may also be an indirect and unintended consequence of migration, resulting in later marriage for girls who had migrated compared to those who had stayed in the village. This perception surfaced more among adults than adolescents but was raised in younger and older groups alike.

*Yes*, *of course migration influences [marital timing]*. *If a girl is engaged and she migrates*, *you who are her father*, *you cannot bring her to her husband as she is not back and some girls are gone two years*, *others even three years before coming back*. *So the moment when she should marry passes*.

Focus group, fathers, Segou

Similarly, respondents reported that labor migration was often directly motivated by girls’ desire to build their *trousseau*. In IDIs with adolescent girls and FGDs with adolescent girls and parents alike, the desire to build a *trousseau* was viewed as a goal and purpose of migration that is inextricably linked with marriage. Respondents reported a girl’s need to look beyond her village for expanded economic opportunities, as shown in the quotation below from a currently married, returned migrant girl:

*Here [in the village] there are not many economic activities aside from buying/selling*, *and even that is not always a good market*. *Because buyers do not have anything*, *it is not easy to trade or sell*. *We have economic difficulties which is what causes us to leave for the big cities*.

In-depth interview, out-of-school, married, returned migrant adolescent girl age 17–19, working as a peanut vendor, Segou

For some respondents, the idea of a girl earning money to build a *trousseau* was an option, but for many, it was an economic necessity, as shown in the following quotation from a married girl who did not migrate because her father told her he would disown any of his girls who migrated:

*The village residents know that migration is not a good thing*, *but if you do not have the means [to build a trousseau]*, *you do not have a choice*. *You will let your girl leave*. *She will work in search of her* trousseau, *in search of money for her marriage* trousseau. *If her mother has nothing and her father has nothing*, *it is the children who do the seeking*, *which is why they do not have a choice*.

In-depth interview, out-of-school, non-migrant married girl age 16–19, Segou

On the other hand, some married girl respondents expressed a belief that migration offers a productive way to spend time for girls who do not have immediate marriage prospects and who have stopped their studies. In an IDI with a married girl who left school at the age of 15 and worked as a domestic worker in Bamako, when she was asked what drove her to move to Bamako, she mentioned not having immediate marriage prospects at the end of her studies:

*Not passing onto superior class made it so that I hated school*. *I also went in search of work*. *I was not yet engaged*.

In-depth interview, out-of-school, returned migrant married girl age 16–19, Segou

For some, migration is a long-term move, but for the most part, the FGDs and IDIs alike suggested migration is something that lasts for a period of time until the girl is ready to return home and marry. Marriage prospects influence girls’ reported length of stay in cities. In FGDs to assess norms, there was a perception that for those who found a spouse, city life became indefinite. This theme did not emerge in IDIs due to selection (see limitations section below). For those who were engaged to be married to someone in the village, their length of stay was indirectly influenced by their ability to make a good living and earn enough money to return home. Some stays reportedly lasted several years, and in some cases, initial earnings afforded girls the right to migrate again, as shown in the quotation below from a married girls’ focus group discussion:

B: *It all depends on how much they earn*. *Certain girls stay for 3*, *5*, *even 6 years…*.

C: *Some can stay three years in adventure; after the three years*, *they come back and give all they have to their parents*. *If the parents are ok*, *they leave again for another three years*

Focus group, married girls, Sikasso

### Migration and city exposure allow girls to acquire skills and develop new perspectives on marriage

Girls acquired skills via domestic work in the city and observed ways of living that influenced their own preparation for marriage. Because girls who migrate may have limited social networks in receiving communities, employers may play a significant role in their development and in determining which skills and knowledge they acquire during their stay. We inquired about the role of a migrant girl’s employer in her development, and the effect of any lessons she may have learned from her employer on her interpersonal skills, and their potential influence on her subsequent trajectory. Engaged and married adolescent respondents and parents reported that a girl’s employer teaches her skills related to housework as well as other skills that will be useful in her marriage and future relationships. This theme of socialization in an urban home as an indirect consequence of migration was reiterated in FGDs with non-married girls, suggesting it may be a normative point of view about the role of the employer in the girl’s development. Most parents did not mention this as a motivation for migration, but rather as an indirect consequence of allowing their girls to migrate. A migrant girl’s relationship with her employer can have a positive or negative influence on how she is perceived upon her return home, as shown in the following quotation from a focus group with mothers in which the facilitator asked a follow-up question about how Bamako helped shaped girls’ experiences:

*Certain girls do not know their obligations to their parents*, *so when they arrive at an employer who educates her children well and the girl learns the good way*, *when she returns she understands respect for family members*, *and they change so much that even the parents ask for and say ‘ah*, *this girl has changed in the good sense; before she was like this and now she is like that*.*’ But if they end up with an employer who cares only about whether the work gets done*, *even if the girl is out all night…in this case*, *if the girl is used to this life of doing what you want and letting things go*, *she is not exemplary here*.

Focus group, mothers, Segou

With changing practices in marriage preparation and an often short duration between return from migration and marriage, parents and adolescent girls perceive that the role of the employer is of great importance in the girl’s development and how she behaves in her new conjugal home. When asked who taught them specific skills in the city, girls referred specifically to their employers, and in a few instances used words like “support” to describe the employer’s influence over the girl. The following quotation from a focus group with fathers demonstrates the belief among parents that in some ways, the employer and his or her values can have a greater influence on the girl than parents in the period shortly before her marriage. In the context of a discussion on the former practice of elders offering advice to girls before marriage in the villages, a perception surfaced about how migration has shifted the sphere of influence on girls’ marital readiness to employers:

*In the past*, *girls’ marriage preparation was very serious here for us but now if you give a girl to a man*, *she leaves in migration*, *and certain girls do not return until one week before their marriage*. *So you cannot educate her anymore with your values*, *but it is more the values of the person for whom she works that guides her; therefore what she does in her husband’s family does not really depend on you*.

Focus group, fathers, Segou

Although the above quotation does not make explicit reference to the type of employer being discussed, later in the FGD participants mentioned domestic duties learned from an employer, so we can infer that the influential employer may be a domestic employer. It is worth noting that our codebook included four sub-codes for type of employment in receiving communities—babysitting, trade, restaurants, and domestic work—and domestic work was the most frequently used sub-code among them. The literature on migrant girls also shows the pervasive nature of domestic work [2].

### Perceived risks of migration and indirect consequences on marriage timing

Respondents identified potential risks of seeking employment in the city. Parents mentioned financial risks including not finding a good job and having to move around a lot, or not making enough money to pay for one’s *trousseau*, as shown in the following quotation from a focus group with girls’ mothers:

*Some girls do nothing but go on walks [*dates*]*. *They do not get settled in a family for work*. *They change employers at every occasion and so cannot amass enough money*. *They experience difficulty when they cannot bring their* trousseau *to the village…But if you stay with a single person*, *when it is time to return [to the village]*, *you will smile and your parents will be fulfilled*.

Focus group, mothers, Sikasso

Some of the other risks and security concerns of city life voiced by adolescents and parents alike were thought to affect marital timing and experience. Examples of this include girls becoming pregnant, either due to abuse by male employers or family members, or girls finding male partners in the city. This predicament was thought to either precipitate marriage or decrease a girl’s chances of marrying altogether. The following quotation from a married girl shows the migration, pregnancy, and martial refusal cycle that her father ingrained in her mind:

*Some migrant girls come back [to the village] with children and won’t accept getting married and no longer accept living in the village*. *I learned of the importance of these ideas from my father*.

In-depth interview, out-of-school, non-migrant married girl, Segou

### Perceived differences between migrants and non-migrants on marriage preparation and marital relations

In a context where marriage and the composition of one’s *trousseau* is often dependent on a girl’s own economic gains, migration was thought to be materially profitable for girls as they could either purchase items in the city or save to purchase them upon return to the village. Mothers and adolescent girls were slightly more likely to describe migration as materially profitable for girls, though the theme surfaced in FGDs with fathers as well. The following quotation from a focus group with unmarried girls shows the perception that migration affords a girl the chance to gather wares for marriage and amass money to purchase *trousseau* items:

*Q*: *What advantages does the [city] adventure bring to [girls]*?

*F*: *Certain girls obtain wardrobes*, *others chairs and many cooking utensils and furniture*.

*C*: *If you have earned money in the city*, *the girl can purchase* trousseau *items here [in the village]*.

Focus group, unmarried girls, Segou

When asked what distinguished migrant and non-migrant girls, some adult focus group participants and some married adolescent interviewees mentioned migrant girls’ *trousseau* as being greater than that of non-migrant girls, while this theme did not surface in many discussions with unmarried girls. The following quotation from a fathers’ FGD shows the perception that the *trousseau* is greater among migrants compared to non-migrants:

*It is the* trousseau. *The one who left for the city will have a greater* trousseau *than the one who stayed here [in the village]*.

Focus group, fathers, Sikasso

It was not clear if a greater *trousseau* resulted in greater agency in deciding whom to marry and/or greater negotiating power within the marriage. In the same discussion, participants raised the perception that migrant girls show more respect toward their husbands and have been exposed to various modes of education compared to non-migrant girls:

*A*: *The difference is respect*. *The one who traveled is more respectful than the one who stayed here; she will respect her husband more than the one who stayed here*.

*B*: *If they go to the big city they learn other modes of education that are not the way of doing things here*. *They can spend a lot of time before marrying compared to others*.

Focus group, fathers, Sikasso

Fathers, engaged girls, and married girls also expressed a belief that observing urban life led migrant girls to learn new skills and behaviors about how to conduct themselves—such as how to walk or sit down properly—and how to relate to others. The development of these skills and behaviors can influence their marriages and relationships with in-laws when they later marry, as shown in the following quotation from a returned migrant married girl:

*The fact of having left for Bamako showed me many things*, *such as*: *how to take care of a husband and mother-in-law*. *I learned these things little by little; all that I did not know how to do I learned there*.

In-depth interview, married, returned migrant girl age 16–19 who never went to school, Sikasso

Other respondents (mostly adults) were less positive and expressed the idea that returned migrants were not fulfilling typical marital tasks for a woman. Women spoke vaguely about challenges migrant girls faced in marriage compared to non-migrant girls (about migration “putting ideas into her head” and “not having the same ideas [as non-migrant girls]” about success in marriage). Conversely, men were somewhat more explicit about the perceived negative effects of migration on marriage. The following quotation from another focus group with fathers expresses a view that returned migrant girls try to live too much like people in Bamako and think they are superior to those who stayed home, thus introducing problems into their marriage:

*Those that leave rural areas in mass come back here with problems…This person is emancipated*. *She can have important sums of money without physical effort…If you stay in the city too long*, *when you come back it is difficult to have a husband here because the person can no longer do physical work as we wish…She thinks she is emancipated*. *You see that she is a woman but if you take her in marriage*, *it is a man that you have because she cannot do all of the domestic chores*.

Focus group, fathers, Sikasso

### Migration allows girls to see different marriage practices

Migration may mean a girl’s first extended time away from her family and in an urban area and can expose her to different ways of doing things. Across respondent groups, migration was believed to allow girls to better understand different roles for women including having fewer domestic responsibilities. Some respondents indicated that this was a positive effect of migration while others cited that migrant girls became stubborn. A focus group of married girls in Segou noted the differences between some women in the city who ‘do nothing’ and the role of women in the village:

*You will find that in the big cities*, *there are certain women who do nothing*: *it is the servant that does all of their work while the women do nothing… You who come from the village*, *you observe how that woman takes care of her husband*, *you take care of her children*. *You put the sheets on the bed*. *You do all that she should do when you go to your own house you cannot be like the girl who has not traveled because you saw a lot of things*.

Focus group, married girls, Segou

For some respondents, the migration experience led girls to develop a different impression of themselves and to reject marriage upon their return to their home villages, thus coloring previous impressions of acceptable futures. This may also reflect new knowledge and skills that are valuable on the marriage market and change a girl’s calculus for choosing an acceptable partner. However, it is worth noting that the following quotation from a focus group with mothers does not capture the perspective of girls who had migrated but did not return to their villages:

*Some girls also when they are engaged go to the big cities*, *and if they succeed*, *upon their return they refuse to marry their fiancé because they believe that they themselves have grown up and have become more beautiful*.

Focus group, mothers, Segou

Fathers echoed the belief about migration and associated economic gains influencing a girl to form a different impression of herself and refuse marriage:

*B*: *The mother says to the boss to pay her the* trousseau *with the money the girl earned during migration*. *This falls on the back of the father*. *The girl returns to her family with it*. *This girl will have difficulty listening to her father*…

*Q*: *Meaning that the material goods that she brings constitute a problem for you*?

Common response: Yes

*Q*: *Meaning that men oppose girls’ migration that is initiated by mothers*?

*Common response*: *Yes*

*B*: *Yes because the material goods she brings to the household are the same material gains at the basis of destruction of marriage (divorce)*.

*Q*: *How*?

*B*: *The material goods that she owns*, *you’ll find that the husband doesn’t have the means to pay for that*. *Two to three days after you’ll see that she no longer respects her husband because she sees only these material goods*, *even if it’s a love marriage*. *So the woman needs to be happy with what her husband has to contribute to the household*.

Focus group, fathers, Sikasso

There were mixed responses on the extent to which migration was thought to influence bride price, or the payment from the groom’s family to the bride’s family. Some respondents believed migration could increase the requested amount of bride price, even if the higher bride price was ultimately not possible. In one FGD, mothers of adolescent girls observed differences between bride price norms in cities and villages and men’s ability to pay and suggested that girls are cognizant of these bride price practices:

*B*: *When [girls] go to the cities*, *what they see*, *that is what creates a lot of difficulty here*. *Marriages in the city are different than marriages here*. *For example*, *if you go to the city*, *we say that the bride price is 150 000…200 000 FCFA*. *But you ask the same thing to a man here [in the village] even though he does not have it*. *That’s a problem*. *He does not have a job that allows him to have this sum of money*. *So he gives a part*, *in explaining that he will have the rest of the money the following year*, *and in the end*, *this remaining amount is not given*.

Focus group, mothers, Sikasso

While girls perceived that migration affected *trousseau*, they were unsure of whether migration had an effect on bride price amount. The following focus group with mothers expressed a different view—that migration had no influence over bride price—as the norms were set by the fathers and not the girls:

*Q*: *Are there also differences between [migrant and non-migrant girls’] bride price*?

*C*: *No*, *there are no differences between the bride price because it is the same fathers who fix their bride price so there are not any differences*.

Focus group, mothers, Sikasso

Girls may believe migration has more influence on *trousseau* in part because their experience of migration may influence girls’ feelings of control over their lives. Girls who migrate may have different expectations about their marriages and their value in the market may increase due to skills and knowledge acquired. Bride price, which is outside girls’ control, may therefore not matter much to girls’ themselves and how they think about marriage. Indeed we heard it did not vary much between those who migrate and those who do not as amounts were established by tradition.

In addition to its influence on timing and preparations for marriage, migration was also perceived by many respondents to influence the type of marriage ceremony that is conducted. Respondents like the father featured in the quotation below explained that migrants are often more likely to want a civil ceremony, that is, a ceremony conducted by a legal official and registered by the court at City Hall rather than a religious or traditional ceremony, although other groups did not observe differences in types of ceremonies for migrant and non-migrant girls:

*There is a very big difference [between those that went to the big cities and those who stayed here]*. *Before*, *there were no civil marriages*. *[Those who migrated] brought that [back] and if in our day the civil ceremony does not happen*, *people say the marriage did not happen*. *It is school and travels that changed that*.

Focus group, fathers, Sikasso

Migration was also thought to influence marriage traditions and the girl’s role in challenging traditional marriage norms. This can create tension between parents and daughters regarding expectations. The following quotation from a focus group with mothers shows that getting one’s hair done for a wedding was a changing marriage practice precipitated by returned migrant girls:

*F*: *Those who have migrated*, *when it comes to marriage*, *they want to have their hair done*. *They want to have everything done*. *Coming back from the city*, *they demand that the marriage is celebrated as in the city*. *If that is not respected*, *they refuse to marry*.

*Q*: *It is necessary that the marriage is done as in the city*?

*F*: *They are no longer respectful of marriage as in the past*. *Many new marriage practices are coming here*. *In the past*, *we did not do a girl’s hair [for her wedding] but now we do*. *It is because of the girls who went to the city that this practice is becoming customary*.

Focus group, mothers, Sikasso

Even if girls returning from urban areas may not be able to avoid marriage, these observed changes in types of marriages or in marriage-related hair and dress fashions may reflect a symbolic challenge to marital traditions and expectations. These changing norms suggest that girls may be able to exert a small but increasing amount of influence in the marital process. Despite issues between parents and daughters regarding marriage, the above quotation demonstrates that there is some middle ground with some ‘city’ traditions being integrated into marriage practices back home.

## Discussion

The present study draws on views of several types of respondents from rural sending communities near Bamako—mothers, fathers, married girls, unmarried girls, engaged girls—to examine the role of adolescent girls’ migration on marriage in Mali, a country where adolescent migration and early marriage are both prevalent. Our findings provide recent evidence that builds on the work of others who have examined migration in Mali, including adolescent migration [9–11] to explicitly explore how adolescent girls’ movement is directly and indirectly linked to four related aspects of early marriage: marital timing, marriage preparations, marriage practices, and marital relations. Our findings also provide important knowledge for programs working with adolescent girls in rural communities in Mali.

DHS data have shown that marriage before age 18 is common in Mali; we suggest that adolescent migration and early marriage are linked in important ways. It is clear that migration presents many opportunities for girls to pursue labor- and education-related goals. Migration to the city affords girls the chance to earn income and gain skills that are valuable both to the composition of a *trousseau* and to domestic life as a wife and mother. Saving for marriage has been observed in other contexts, including Egypt and Bangladesh (see, for example [23, 24]). Our study contributes to the literature on delayed marriage in sub-Saharan Africa by focusing on women and girls’ economic obligations in marriage, such as *trousseau*, rather than focusing solely on bride price requirements of men.

Our findings indicate that migration influences numerous aspects of marriage: marriage timing, marriage preparations including development and value of the *trousseau* and bride price amount, marriage practices including types of marriage ceremonies and hair and dress traditions, and martial relations including either marriage refusal or understanding roles for men and women in marriage. As individuals form perceptions of migration—regardless of whether or not they themselves had migrated—they are making meaning of marriage expectations and social norms about marriage in their community.

Findings from this research suggest that girls who migrate may bring back not only items or income that are tangible and intended benefits, but also different viewpoints on the expectations for women and girls in their communities. Although this can be challenging for returned girls in terms of reintegration, these new expectations may lead to social changes over time. Migration also offers girls the opportunity to visualize alternative pathways for their futures, which may have positive or negative consequences for them. It stands to reason that for girls who migrate, acquisition of new skills and exposure to different values may have the potential to ultimately influence partner selection, but further research is needed on this topic. Previous research on transfers from the bride’s family (dowry) in other contexts supports the claim that girls with more substantial dowry may be able to attract partners of higher, or least equal, social standing [25]. However, despite some of migration’s positive effects on girls [1, 2], there are still risks associated with migrating.

There are some study limitations that temper our findings. This analysis draws heavily on inputs from FGDs with mothers and fathers, as adult respondents proved more likely than adolescent girls to share information and expand on key discussion points. We also acknowledge that additional probing may have yielded more insight from some of the younger respondents. Budget limitations prevented us from gathering perceptions from adolescent boys. Moreover, the quantitative baseline study showed a mean age difference of 10.4 years between adolescent girls and their marriage partners [17], suggesting that a complete consideration of the perspectives of partners would have to include a wide age range of men. Future research would benefit from their perspective in understanding how young men are conditioned to think about adolescent girls’ migration and its perceived impact on marriage.

Another study limitation is that the samples were drawn from MTBA program intervention areas, which may have greater transport networks and may have higher rates of out-migration than other areas in Mali. Among migrant girls, only those who had returned to their sending communities were included in our sample, so we were not able to capture the perspectives of girls who had migrated more permanently. Future research on marriage and migration should include samples drawn from both sending and receiving communities. In addition, respondents may be more inclined to speak of migration as a way of pursuing opportunities (e.g. work to gain her *trousseau*) than as a way of avoiding hardship at home, thus biasing responses toward more favorable factors.

Future quantitative research with adolescents in Mali would benefit from including attitudinal questions on expectations related to marital practices and relations (e.g. types of ceremony desired, types of tasks married women should perform) and should look for potential differences among those who had migrated and those who had not. Future quantitative studies could also use life event history questions to understand duration of migration, patterns of seasonal or step migration, and duration between migration and marriage. In areas where early marriage is prevalent, qualitative research is needed to shed light on the effects of girls staying in school and unmarried on marriage norms.

Given how pervasive adolescent migration appears to be in these communities, programs need to take a multi-pronged approach in reaching girls along the migration trajectory. First, in sending communities, programs to delay marriage may consider enlisting returned migrant girls as early adopters [26] who can serve as role models for other non-migrant girls who want to delay marriage. Programs can focus on equipping girls with skills, such as communication and negotiation abilities, and the ability to manage money and save, that will be useful whether girls stay in cities or move home and marry. Innovative program supports like visual toolkits can also reinforce content covered in interventions so girls who move have information that moves with them and potentially the ability to teach this content to others. In cities and migration receiving communities, interventions can build on the influence of employers, as reported in this study, to teach girls skills that prepare them for their conjugal life, and how to avoid high risk behaviors and access resources while living away from home. Likewise, given anecdotal observations about seasonal and circular migration patterns in Mali and elsewhere in West Africa, interventions can explicitly design program duration around these movement patterns so that program participants have the opportunity to be exposed to all program content before potentially moving onto a new migration destination or returning home.

## Supporting information

S1 Appendix List of codes used in this analysis. (DOCX)
