# Supplementary material for: Adolescent girls’ migration and its impact on early marriage: Qualitative findings in Mali
Source: PLoS One. 2020 Mar 20;15(3):e0230370. doi: 10.1371/journal.pone.0230370 (PMC7083269; doi:10.1371/journal.pone.0230370)
Supplement: S1 Appendix — (DOCX) [file pone.0230370.s001.docx]

**S1: Appendix: List of Codes used in this Analysis**

| **Reasons for migration decisions** |  |
| --- | --- |
| Reasons for migration | Includes reasons the girl herself had or reasons others had for her. |
| Avoid marriage | Includes whether marriage avoidance is due to age or selection of partner. |
| Work/gain her trousseau |  |
| Done with school and need to do something |  |
| Influence of return migrants | How her perception of return migrants shapes her own migration desires |
| Family dynamics | Includes family influences at home that push girl to migrate (e.g. illness or death of a parent) or family influences that pull girl to migrate to the city (e.g. success in getting a good job in city). |
| Reasons for not migrating |  |
| Help with household chores at home |  |
| In school |  |
| Need to care for sick family members |  |
| Bad reputation of migrant girls | Parents refuse to let girls migrate due to their behavior (frivolous, lazy, refusing marriage upon return, not listening to parents) |
| **Non-migrants’ perceptions of migration** |  |
| How migrants conduct themselves in the city |  |
| How migrants conduct themselves upon return |  |
| **Work and living arrangements in city** |  |
| Who she lives with | Includes either family members or employers; also includes who she stayed with initially upon arrival as relevant. |
| Risks and safety | Includes risks girls face and threats to their safety while living in the city. |
| Types of job |  |
| Domestic work | Refers to all tasks related to housework for a host family. |
| Babysitting | Includes caring for other people’s children or a relative. |
| Commerce | What she sold and where she sold it. |
| Restaurant |  |
| **Migration trajectories** | How respondents define migration (i.e. a particular duration); includes cases where they originally report not living elsewhere but when interviewer prompts them with a follow-up question, they then report having lived elsewhere. |
| Age of migration | Includes ages of initial migration and subsequent migrations as relevant. |
| Destinations | Includes destinations within Mali and those in neighboring countries. |
| Number of trips made | For repeat migrants (whether seasonal or circular), this code quantifies the number of trips made. |
| Step migration with smaller cities first | Includes initial destinations and subsequent destinations for those who made multiple trips. |
| Duration of trips | Includes how long she stayed in each place. |
| Seasonality of migration | Refers to timing of year when she made trips and how it may relate to rainy and/or dry season. |
| **Influence of the city on the girl** |  |
| Social networks | Who was in the migrant girl’s network and how often she saw them. |
| Mobility in the city | Key places in the city where migrant girl went and with what regularity. |
| Reproductive health knowledge | What migrant girls learn and who they learn it from. |
| Behaviors learned that are useful in marriage | What she learned and who she learned it from. Includes things like how to obey your spouse, how to get along with your in-laws. |
| Household skills learned that are useful in marriage | Includes things like cooking, laundry, how to take care of a child. |
| Role of patronne in her learning | What she learned by observing patronne and advice given. |
| Media | Includes her access to media and the content. |
| Telephone | Telephone purchase, people with whom she is in contact thanks to the phone, credit costs, types of use, etc. |
| **Migrant’s earnings/trousseau and financial behaviors in city** |  |
| Amount earned | Includes type of job and amount earned weekly or monthly. |
| Savings mechanism |  |
| Employer keeps money |  |
| She keeps money |  |
| Someone else in city keeps money |  |
| Money sent home |  |
| Items in trousseau purchased |  |
| Expenditures | What she used her earnings for in city |
| Perceptions of banks | Attitudes towards banks and formal savings mechanisms and appetite for them. |
| **Influence of migration on** |  |
| Bride price amount |  |
| Desired age of spouse |  |
| Marital refusal |  |
| Marital relations | Includes ability to get along with spouse and related power dynamics. |
| Marital timing |  |
| Earlier for migrants |  |
| Later for migrants |  |
| No difference for migrants and non-migrants |  |
| **Attitudes toward timing of marriage** | Note this code is tied in with the effect of migration on marriage. |
| Advantages of earlier marriage |  |
| Avoid girls misconduct | Includes walks (dates) with boys, pregnancy outside of marriage, and subsequent family shame. |
| Advantages of later marriage |  |
| Effects on pregnancy and delivery |  |
| Ability to do arduous housework |  |
| Acquisition of additional skills |  |
| Ability to get along with others | Includes husband, in-laws, and co-wives. |
| **Differences between migrants and non-migrants** | Including the response that there is no difference between migrants and non-migrants. |
| Earnings |  |
| Material objects | Includes objects that are part of trousseau and other items. |
| Literacy | Differences in ability to read and write and when these skills are needed. |
| Learned behaviors | How to get along with others, how to conduct herself with respect. |
| Access to information | Includes sexual and reproductive health (SRH) information and other information. |
| Type of marital ceremony | Influence of migration on decisions relating to type of martial ceremonies (religious, civil, traditional). |
| Perspective on her own life and how others live |  |
